# Pressure-Induced Alterations in PEDF and PEDF-R Expression: Implications for Neuroprotective Signaling in Glaucoma

**DOI:** 10.4172/2155-9570.1000491

**Published:** 2015-10-27

**Authors:** Sean J Lee, D’Anne S Duncan, Franklin D Echevarria, William M McLaughlin, Jeremy B Hatcher, Rebecca M Sappington

**Affiliations:** 1 Department of Ophthalmology and Visual Sciences, Vanderbilt Eye Institute, USA; 2 Department of Pharmacology, Vanderbilt University Medical Center, USA

**Keywords:** Neuroinflammation, Glia, Retinal ganglion cell, Pigmented epithelial growth factor, Glaucoma, Microbead occlusion model, Apoptosis

## Abstract

**Introduction:**

Alterations in neuron-glia signaling are implicated in glaucoma, a neurodegenerative disease characterized by retinal ganglion cell (RGC) death. Pigment epithelium derived factor (PEDF) is a secreted protein with potential neuroprotective qualities in retinal disease, including chronic ocular hypertension. Here we sought to determine whether moderate, short-term elevations in IOP alter PEDF signaling and whether pressure-induced PEDF signaling directly impacts RGC apoptosis.

**Methods:**

In retina from naïve mice and mice with unilateral, microbead-induced glaucoma, we examined expression and cell type-specific localization of PEDF and its receptor (PEDF-R), using quantitative PCR and immunohistochemistry. Using primary cultures of purified RGCs and Müller cells, we examined cell type-specific expression of PEDF in response to 48 hours of elevated hydrostatic pressure, using multiplex ELISA and immunocytochemistry. We also measured pressure-induced apoptosis of RGCs in the presence or absence of atglistatin, a potent and selective inhibitor of PEDF-R, and recombinant PEDF, using TUNEL assays.

**Results:**

PEDF and PEDF-R are constitutively expressed in naïve retina, primarily in the ganglion cell and nerve fiber layers. Elevated IOP increases PEDF and PEDF-R expression, particularly associated with RGCs and Müller cells. Elevated pressure *in vitro* increased PEDF secretion by 6-fold in RGCs and trended towards an increase in expression by Müller cells, as compared to ambient pressure. This was accompanied by changes in the subcellular localization of PEDF-R in both cell types. Inhibition of PEDF signaling with atglistatin increased pressure-induced apoptosis in RGCs and treatment with recombinant PEDF inhibited pressure-induced apoptosis, both in a dose-dependent manner.

**Conclusion:**

Our findings suggest that moderate, short-term elevations in IOP promote PEDF signaling via up-regulation of both PEDF and PEDF-R. Based on *in vivo* and *in vitro* studies, this PEDF signaling likely arises from both Müller cells and RGCs, and has the potential to directly inhibit RGC apoptosis.

## Introduction

Glaucoma is a neurodegenerative disorder that leads to irreversible vision loss via the death of retinal ganglion cells (RGCs) and their axons, which form the optic nerve. Glaucomatous neurodegeneration is associated with both age and sensitivity to intraocular pressure (IOP), which are the two primary risk factors for the disease [1,2]. A myriad of cell stressors and pathological events are involved in glaucoma, including ischemia, excitotoxicity, neuroinflammation, and neurotrophic factor deprivation [1,2]. Glial cells are implicated as mediators of several of these processes via expression of pro-inflammatory cytokines and chemokines, as well as neurotrophins [3,4].

Recent evidence suggests that pigment epithelium-derived factor (PEDF) may be neuroprotective for RGCs in both *in vitro* and *in vivo* models of glaucoma, including the DBA/2 model of chronic glaucoma [5,6]. While PEDF is typically classified as an anti-angiogenic factor that is produced by epithelial cells, PEDF also appears to perform neurotrophic and anti-inflammatory functions [7–9]. In retina, PEDF promotes differentiation of precursor cells into cells with neuronal phenotypes [10] and reduces Müller cell activation in response to glaucoma-related stressors [11]. Recent studies confirm that PEDF is expressed in particularly high concentrations by Müller cell end-feet, which are in close proximity to RGC cell bodies and their unmyelinated axons in the ganglion cell and nerve fiber layers, respectively [12–14]. Furthermore, the high-affinity receptor for PEDF (PEDF-R) localizes to RGC somas [15]. As such, it appears that Müller cell-derived PEDF may be most relevant for neuroprotection of RGCs in glaucoma [6].

While sufficient evidence suggests that PEDF, particularly Müller cell-derived PEDF, may be relevant for RGC survival in glaucoma, it is unclear how glaucoma-related stressors specifically modify constitutive PEDF signaling. Moreover, previous studies have not examined the precise role of pressure, the only modifiable glaucomatous risk factor, on PEDF signaling [5,6]. As such, the objective of this study was to: 1) characterize constitutive and pressure-dependent expression of PEDF and PEDF-R by specific retinal subtypes and 2) determine the direct impact of PEDF signaling on pressure-induced RGC apoptosis. Using quantitative PCR, multiplex enzyme-linked immunosorbance assay (ELISA) and immunohistochemistry, we determined global and cell type-specific expression of PEDF and PEDF-R in retina from naïve mice and mice with microbead-induced ocular hypertension [16]. Cell type-specific expression was further confirmed in primary cultures of purified Müller cells and RGCs. Lastly, we measured apoptosis in RGC cultures exposed to ambient pressure or elevated hydrostatic pressure in the presence or absence of the PEDF-R inhibitor atglistatin and recombinant PEDF.

We found that constitutive expression of PEDF and PEDF-R preferentially localized to the inner retina. Interestingly, PEDF and PEDF-R were expressed by both Müller cells and RGCs. This pattern of localization was retained in retina from both saline- and microbead-injected eyes. However, the level of expression for both proteins increased significantly in glaucomatous retina. *In vitro* studies further confirmed that both RGCs and Müller cells increase expression of PEDF and PEDF-R in response to elevated pressure. These data suggest that PEDF can act as both an autocrine and a paracrine signal within and between RGCs and Müller cells in retina. Furthermore, that RGCs and Müller cells upregulate both PEDF and PEDF-R in response to elevated pressure suggests a role for both autocrine and paracrine pathways in cellular responses to glaucoma-related stressors. And lastly, PEDF-R inhibition induces RGC apoptosis, only to be exacerbated in elevated pressure conditions. Together, our data indicate that PEDF signaling in glaucoma is more complex than initially appreciated, and that the neuroprotective effects of PEDF could arise from multiple pathways of PEDF signaling within and between RGCs and Müller cells.

## Materials and Methods

### Animals

This study was conducted in accordance with the regulations of the ARVO Statement for the Use of Animals in Ophthalmologic and Vision Research; the Animal Care and the Use Committee of Vanderbilt University Medical Center approved all protocols. Male C57BL/6 mice (n=19) were obtained at 4 months of age from Charles River Laboratories (Wilmington, MA). For primary cultures, timed-pregnant rats were also obtained from Charles River Laboratories (n=6 dams, n=64 pups). The animals were maintained by the Vanderbilt Department of Animal Care in a 12 hour light, 12 hour dark cycle with food and water available ad libitum.

### Microbead occlusion model

To induce 4 weeks of ocular hypertension in mice, we performed microbead surgeries as previously described [16]. Briefly, animals were anesthetized with isoflurane (Minrad Inc., Bethlehem, PA), and pupils were dilated with 1% tropicamide ophthalmic solution (Bausch & Lomb, Tampa, FL). Using a pulled-glass micropipette and microinjection device, one eye was injected with 1.5 µL sterile 15-µm polystyrene beads in saline (1×10^6^ microbeads/ml; Invitrogen; Eugene, OR). The contralateral eye was injected with an equivalent volume of sterile physiologic saline (Fisher Scientific, Fair Lawn, NJ). After injection, antibiotic drops (0.5% moxifloxacin HCl; Alcon, Fort Worth, TX) were placed on each eye. IOP measurements were obtained with a tonometer (TonoLab, Icare; Raleigh, NC) on unanaesthetized animals, as previously described [4,17]. IOP was determined as the mean of 10 individual measurements. Baseline IOP readings were recorded for 3 consecutive days prior to microbead or saline injections. Following microbead and saline injections, IOP readings were recorded 3 times per week for 4 weeks, when the experiment was terminated.

### Tissue preparation and harvest

For analysis of fresh retina tissue, mice were sacrificed by cervical dislocation followed by decapitation. Whole eyes were enucleated, flash-frozen and stored at −80°C until RNA and protein isolation. For tissue procurement for paraffin-embedded and whole-mount retina analyses, mice received an overdose of pentobarbital (200 mg/ kg; Hospira, Inc., Lake Forest, IL) and were sacrificed by transcardial perfusion with phosphate buffered saline (PBS; Fisher Scientific; Pittsburgh, PA) followed by 4% paraformaldehyde (PFA; Electron Microscopy Sciences, Hatfield, PA). Whole eyes were enucleated and post-fixed for 1 h in PFA. For paraffin retina sections, eyes were paraffin embedded and 6 µm serial sections of the entire globe were obtained. For whole-mount preparations, retinas were dissected from eyecups and the vitreous was removed.

### Quantitative real-time polymerase chain reaction (qRT-PCR)

Total RNA was isolated from retina, as previously described [3]. Following QC/QA analysis of RNA, reverse transcription of 10 ng RNA was performed to generate cDNA using SuperScript Enzyme Mix and VILO Reaction Mix (Life Technologies, Grand Island, NY) as a dNTP source. Levels of specific cDNA transcripts were assessed using TaqMan Gene Expression Master Mix (Applied Biosystems, Forest City, CA) and 1 µL of TaqMan probes specific for PEDF (Catalog #Mm00441270_m1), PEDF-R (Catalog #Hs00386101_m1), and glyceraldehyde 3-phosphate dehydrogenase or *gapdh* (Catalog #Mm99999915_g1) on a 7900HT Fast Real-time PCR system in triplicate (Applied Biosystems). The Δ-CT method was used to determine gene expression, using *gapdh* as the control gene. All samples were run in duplicate.

### Immunohistochemistry (IHC)

For PEDF and PEDF-R localization studies, we performed immunohistochemistry on paraffin-embedded longitudinal sections of whole eyes, whole-mounted retinas, as well as primary cultures of purified RGCs and Muller cells. For paraffin-embedded tissue, sections were de-paraffinized, rehydrated, and treated with 0.1% NaBH4 (Fisher Scientific) to quench auto-fluorescence. Whole-mounted retinas were cyroprotected in a graded sucrose series and subjected to 3 consecutive freeze-thaw cycles. Primary cultures were fixed for 30 minutes with 4% PFA. IHC was performed as previously described [4,17,18]. Briefly, samples were incubated in blocking solution containing 5% normal horse serum and 0.1% Triton-X in PBS. Sections were then incubated overnight (paraffin/cultures) or 4 days (whole-mount) at 4°C in primary antibody solution containing primary antibodies (Table 1), 3% horse serum and 0.1% Triton X-100 diluted in PBS. Following PBS washes, samples were incubated for 2 hours at room temperature (paraffin/cultures) or overnight at 4°C (whole-mount) in a secondary antibody solution containing 1% horse serum, 0.1% Triton X-100, and the appropriate secondary antibodies – including Alexa Fluro 488-donkey anti-mouse, Rhodamine Red-donkey anti-goat and Alexa Fluro 647-donkey anti-rabbit (1:200; JacksonImmuno, West Grove, PA). Finally, paraffin sections were counterstained with the nuclear stain DAPI (1:100; Invitrogen) prior to cover-slipping in aqueous mounting media (Southern Biotech, Birmingham, AL).

**Figure 1:**
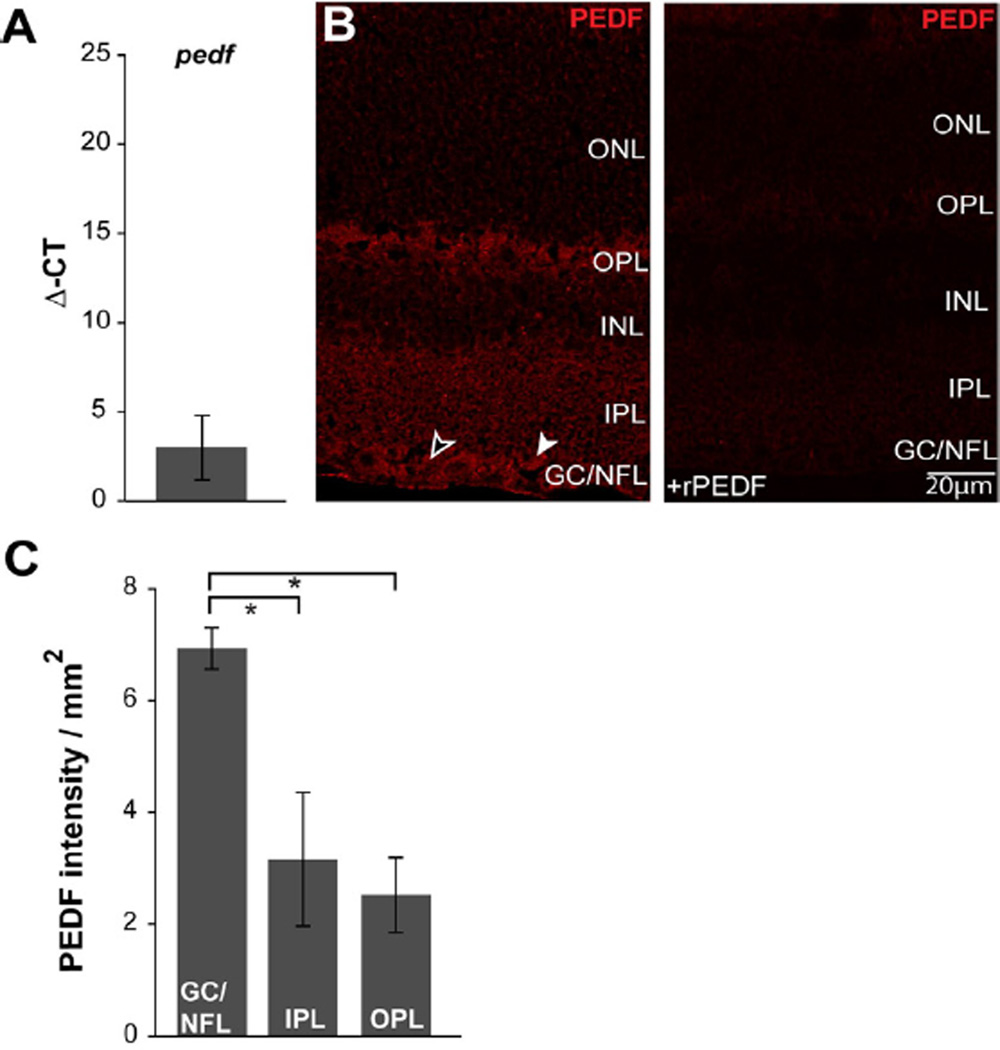
PEDF is constitutively expressed in healthy retina. **A.** Graphical representation of pedf total mRNA levels in naïve C57 retina as measured by qRT-PCR. Y-axis represents the average delta of threshold cycle (Δ-CT) values for pedf normalized to the control gene gapdh. **B**. Representative micrograph of longitudinal sections of retina from naïve mice immunolabeled with antibody against PEDF with (right panel) and without (left) pre-absorption with recombinant PEDF (rPEDF). Morphology consistent with localization to RGCs (white arrowheads) and Müller cells (black arrowheads). **C**. Retinal layer-specific quantification of PEDF labeling, expressed in intensity (arbitrary units) per area (mm^2^). All asterisks denote p<0.05. ONL: Outer Nuclear Layer; OPL: Outer Plexiform Layer; INL: Inner Nuclear Layer; IPL: Inner Plexiform Layer; GCL: Ganglion Cell Layer; NFL: Nerve Fiber Layer.

To confirm binding specificity of anti-PEDF antibody, we performed immunohistochemistry on paraffin-embedded longitudinal sections of naïve retina as described above. Sections were incubated overnight at 4°C in primary antibody solution containing anti-PEDF antibody (Table 1) with Recombinant PEDF protein (20 µg/ml; R&D Systems, catalog #1177-SF-025). Following PBS washes, samples were incubated for 2 h at room temperature in a secondary antibody solution with Rhodamine Red-donkey anti-mouse (1:200; JacksonImmuno). Finally, paraffin sections were counterstained with DAPI (1:100; Invitrogen) prior to imaging (Figure 1).

### Quantification of layer-specific immunolabeling from paraffin sections

To evaluate PEDF and PEDF-R retina layer-specific expression, 40× images were taken using a Roper Scientific black and white camera (Photometrics, Tucson, AZ) mounted to a Nikon Ti microscope (Nikon Instruments, Melville, NY). Regions of interest were outlined around outer nuclear layers (ONL), outer plexiform layers (OPL), inner nuclear layers (INL), inner plexiform layers (IPL), and ganglion cell-nerve fiber layers (GC/NFL) using NIS Elements software (Nikon). From those regions of interest, mean fluorescent intensity per area (arbitrary units/mm^2^) was measured.

### Quantification of PEDF secretion using luminex technology

Supernatants from primary cultures of purified RGCs and Müller cells were collected at the termination of the hydrostatic pressure experiments (48 hours) as previously described [3,19–21]. Using Milliplex MAP kit and Luminex technology, we performed a human-specific PEDF magnetic bead immunoassay (catalog #HNDG2MAG-36K) as per manufacturer’s instructions (Millipore). Human-PEDF has an amino acid sequence homology of over 90% compared to the mouse variant, as determined by online Basic Local Alignment Search Tool (BLAST). Briefly, Müller cells and RGCs supernatants, PEDF standards, and controls were incubated in a 96-well plate with PEDF magnetic beads overnight at 40°C. Samples were then incubated in PEDF detection antibodies for 1 h at room temperature, and subsequently in streptavidin-phycoerythrin for 30 minutes at room temperature. Protein concentrations of PEDF were measured with a multiplex plate reader (MAGPIX with xPONENT software; Millipore), as determined by the PEDF standard curve. All samples were performed in duplicate with a minimum of 4 samples per condition.

### Cell culture preparation

Primary cultures of purified RGCs were prepared from P3 rat pups by immunomagnetic separation, as previously described [3,19–21]. Briefly, retinas were dissociated with papain and trituration. Retinal cell suspensions were then incubated in mouse anti-rat Thy-1.1/CD90 IgG (5 µg/ml; BD PharMingen, San Diego, CA) for RGC selection or mouse anti-rat CD44 (15.625 µg/ml; Novus Biologicals, Littleton, CO) for Müller cell selection. Suspensions were then incubated with metallic microbeads conjugated to anti-mouse IgG1 microbeads (Miltenyi Biotech, San Diego, CA). RGCs and Müller cells were then positively selected in a proprietary column placed in a magnetic field with metallic microbeads conjugated with anti-mouse IgG (Miltenyi Biotech).

RGCs were plated at a density of approximately 3 × 10^3^ cells in each well of eight-chamber glass slides (Labtek 2; Nal-Nunc, Rochester, NY) coated with laminin (0.01 mg/ml; Sigma Aldrich, St. Louis, MO) and poly-D-lysine (0.01 mg/ml; Sigma). Cells were grown in serum-free, B27-supplemented media (NeuroBasal; Gibco, Carlsbad, CA) containing 2 mM glutamine, 0.1% gentamycin, 50 ng/ml brain-derived nerve growth factor (Invitrogen), 20 ng/ml ciliary neurotrophic factor (Invitrogen), 10 ng/ml basic fibroblast growth factor (Invitrogen), and 100 µM inosine (Sigma). RGCs were used for experiments 3–4 days after plating. Müller glial cells were plated at a density of 1×10^5^ in each well of two-chamber glass slides (LabTek) and were grown in 1:1 DMEM/F12 media containing 0.1% gentamycin, 10% fetal calf serum, and 1% G5 supplement (Invitrogen). Confluent chamber slides of Müller cells were used for experiments approximately 10 to 14 days following plating.

### Hydrostatic pressure experiments and PEDF-R Inhibition

Primary cultures of purified RGCs and Müller cells were maintained at ambient pressure in a standard incubator or at +70 mmHg hydrostatic pressure, using a custom-made regulator chamber placed in the incubator, as previously described [3,19–21]. Briefly, a humidified pressure chamber equipped with a regulator and a gauge was placed in a 37°C incubator; a mixture of 95% air and 5% CO_2_ was pumped into the chamber to obtain a pressure of +70 mmHg (9% increase above atmospheric pressure) that was maintained by the regulator. For ambient pressure experiments, cells were kept in a standard incubator. Each experiment was performed in quadruplicate.

For functional PEDF studies, primary RGC cultures were treated with varying concentrations of atglistatin, a selective, potent competitive inhibitor [22] of PEDF-R (Catalog #530151; Millipore) or recombinant PEDF. After RGC plating in 8-well slides, atglistatin was added to culture media in five treatment groups: 0 µM (DMSO alone), 50 µM, 100 µM, 200 µM, and 400 µM. Recombinant PEDF (R&D Systems) was added to the culture media at concentration of 50 ng/ml or 100 ng/ml. RGCs were then subjected to hydrostatic experiments as described above for 48 hours.

### Terminal deoxynucleotidyl transferase-mediated biotinylated UTP nick-end labeling (TUNEL) staining

RGC viability was assessed following the manufacturer’s protocol of the TUNEL apoptosis assay (Catalog #17-141; Millipore) for the detection of endonucleolytic cleavage of chromatin. Cultured RGCs were also subjected to ICC as described above, using RGC-specific markers (SMI-31, β-tubulin, or brn-3a; Table 1) with the appropriate secondary antibodies, and counterstained with DAPI to identify RGCs in culture. TUNEL staining was analyzed using a fluorescent microscope; percent apoptosis was obtained by dividing the number of TUNEL-positive RGCs by the total number of RGCs per section, as determined by co-localization between DAPI and RGC marker.

### Statistical analysis

All statistical tests were conducted with SigmaPlot (Systat Software Inc., San Jose, CA). Experimental groups were compared with one-way ANOVA with pairwise comparisons by Bonferroni post-hoc analyses. All data is presented as mean ± standard deviation, with significant comparisons marked by red brackets and asterisks. For all analyses, p ≤ 0.05 was considered statistically significant.

## Results

### PEDF is constitutively associated with RGCs and Müller cells in healthy retina

We first examined expression and localization of PEDF in retina from naïve C57 mice. In accordance with previous literature [7,8,10], we detected constitutive expression of the *pedf* gene in murine retina (Figure 1A). To determine where PEDF is expressed constitutively, we performed PEDF immunolabeling in longitudinal sections of paraffin-embedded whole eyes. This immunolabeling revealed the presence of PEDF protein in all plexiform layers of the retina, as well as the ganglion cell (GCL) and nerve fiber (NFL) layers. In retina, the intensity of immunolabeling appeared most robust in the GCL and NFL (arrowheads, Figure 1B). In the GCL and NFL, PEDF protein appeared to associate with RGCs, whose cell bodies are present in the GCL (white arrowheads) as well as Müller cell end-feet in the NFL (open arrowheads; Figure 1B). Müller cell end-feet were identified by the triangular processes that extend between RGC soma in the GCL to terminate in the NFL (black arrowheads; Figure 1B). Pre-absorption of our primary antibody with recombinant PEDF protein (rPEDF) competitively inhibited binding of our primary antibody to retina, indicating specificity of our antibody for PEDF (Figure 1B). To verify relative differences in PEDF localization to retinal layers, we quantified the intensity of PEDF immunolabeling in each layer (Figure 1C). Consistent with our qualitative observations, the intensity of PEDF immunolabeling was 2.7-fold and 2.2-fold greater in the GC-NFL than in the outer and inner plexiform layers, respectively (p<0.05 for both; Figure 1C).

**Figure 2:**
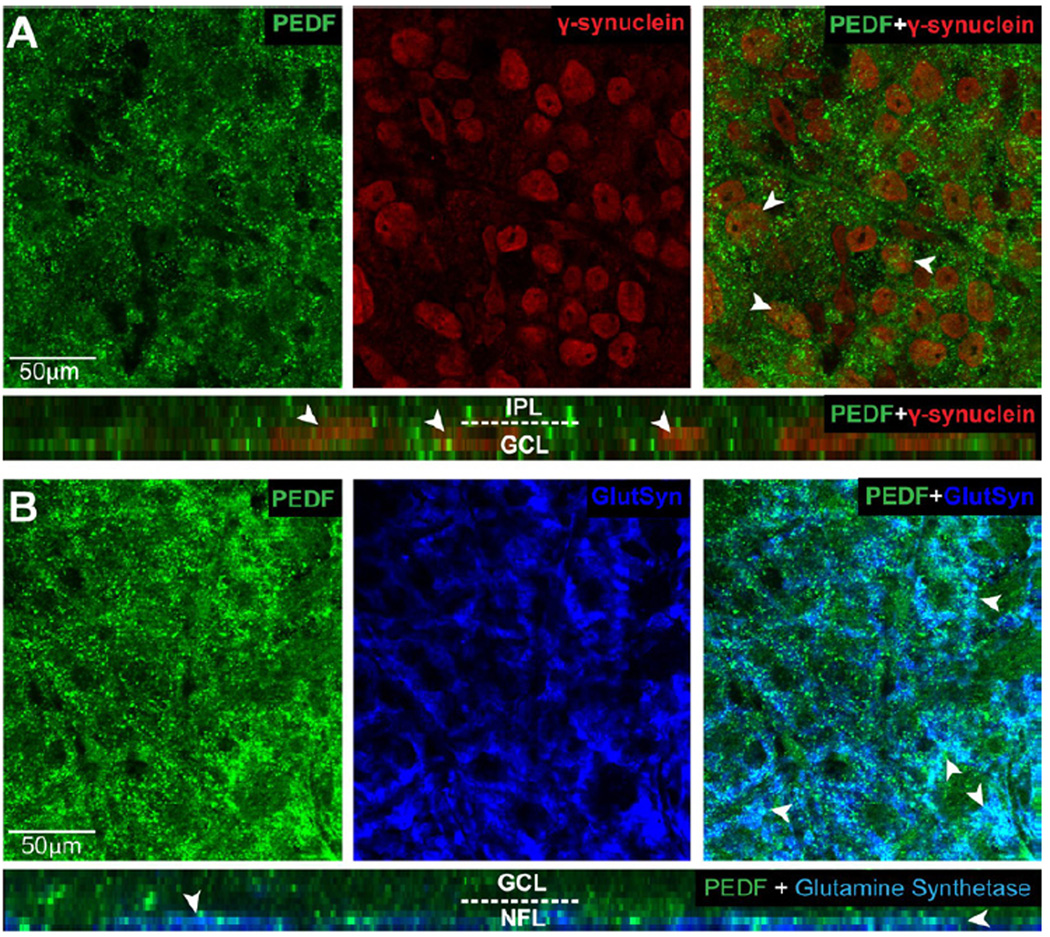
PEDF is associated with RGCs and Müller cell endfeet in the ganglion cell and nerve fiber layers. **A**. Representative confocal micrographs with orthogonal view (bottom panel) of wholemount retina from naïve mice co-immunolabeled with antibodies against PEDF (green) and the RGC marker γ-synuclein (red). PEDF localizes to the area surrounding PEDF γ-synuclein+ RGCs in the ganglion cell layer (GCL; arrowheads). **B**. Representative confocal micrographs with orthogonal view (bottom panel) of wholemount retina from naïve mice co-immunolabeled with antibodies against PEDF (green) and the Müller cell marker glutamine synthetase (GluSyn; blue). PEDF localizes to the area surrounding Müller cell endfeet in the nerve fiber layer (NFL).

To confirm that PEDF associates with RGCs and Müller cells in the GCL and NFL, we performed co-immunolabeling against PEDF and cell type-specific markers for RGCs (γ-synuclein) and Müller cells (glutamine synthetase) in whole-mounted retina from naïve eyes. As expected by the immunolabeling pattern in vertical sections of retina, PEDF immunolabeling was present throughout the GCL and NFL (Figures 2A and 2B). Co-immunolabeling with antibodies against γ-synuclein revealed the presence of PEDF in the extracellular space surrounding γ-synuclein+ RGC soma (Figure 2A). This association was confirmed in orthogonal slices of our confocal z-stacks that illustrate localization of PEDF protein to the GCL in areas surrounding the perinuclear labeling of γ-synuclein in RGCs (white arrowheads, Figure 2A, bottom panel). Similarly, co-immunolabeling with antibodies against glutamine synthetase revealed the presence of PEDF in and around glutamine synthetase+ Müller cell end-feet in the NFL, as evidenced by the white appearance of co-labeling (white arrowheads, Figure 2B). This association was confirmed in orthogonal slices of our confocal z-stacks through the NFL that depict localization of PEDF protein (green) to glutamine synthetase+ endfeet (white) as well as in the extracellular space surrounding glutamine synthetase+ endfeet (blue; Figure 2B, bottom panel).

### Elevated IOP induces PEDF expression in murine retina

**Figure 3:**
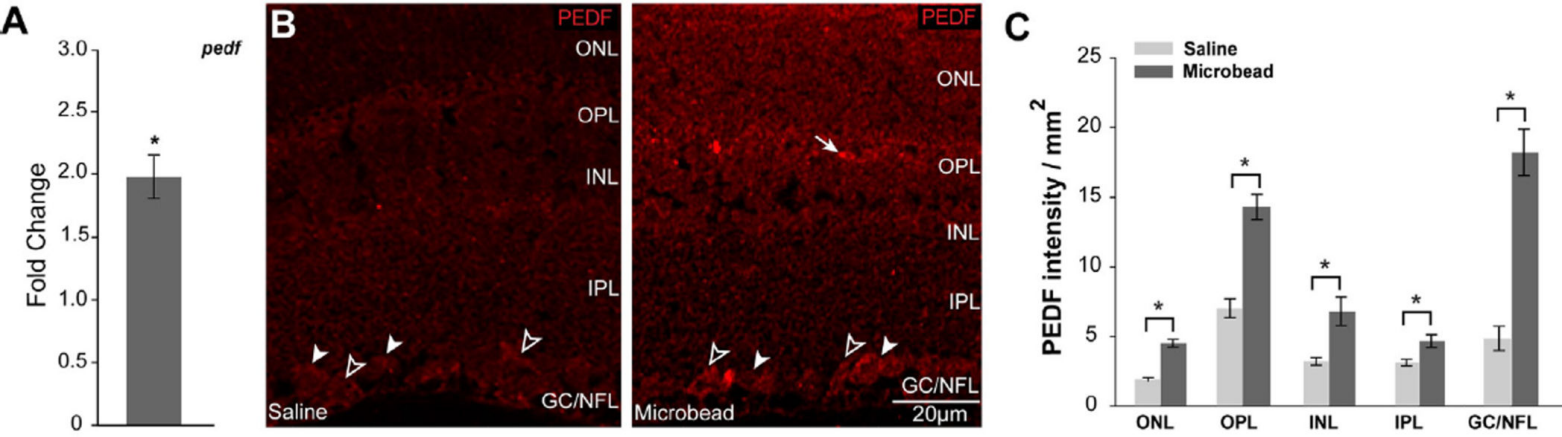
Elevated IOP increases PEDF expression in murine retina. **A**. Graphical representation of changes in pedf total mRNA in retina from saline- and microbead-injected mice, as measured by qPCR. Y-axis represents the fold-change in Δ-CT values for pedf (normalized to the control gene gapdh) microbead-injected, as compared to saline-injected. **B**. Representative micrographs of immunolabeling for PEDF in longitudinal sections of retina from saline- (right panel) and microbead-injected (left panel) mice demonstrate increased intensity of labeling in all layers of retina from microbead-injected eyes, as compared to saline-injected eyes. The pattern of PEDF immunolabeling is consistent with RGC soma in the GCL (white arrowheads) and Müller cell processes and endfeet in the ONL and NFL (black arrowheads), respectively. **C**. Retinal layer-specific quantification of PEDF labeling, expressed in intensity (arbitrary units) per area (mm2). All asterisks denote p<0.05. ONL: Outer Nuclear Layer; OPL: Outer Plexiform Layer; INL: Inner Nuclear Layer; IPL: Inner Plexiform Layer; GCL: Ganglion Cell Layer; NFL: Nerve Fiber Layer

Next, we sought to determine how PEDF expression is altered by elevated IOP, the primary modifiable risk factor for glaucoma. Using the Microbead Occlusion Model, which is characterized by modest elevations in IOP [16,23] without overt secondary inflammation, we examined PEDF expression and localization in mice with 4 weeks of unilateral ocular hypertension. In retina from microbead-injected, hypertensive eyes (mean IOP=20.6 ± 3.1 mmHg), *pedf* mRNA increased nearly two-fold (p<0.05), as compared to retina from saline-injected, normotensive eyes (mean IOP=14.8 ± 1.9 mmHg; Figure 3A). There was no significant difference in retinal gene expression of *pedf* between naïve and saline-injected conditions (p>0.05).

To determine where in the retina IOP-induced expression of PEDF occurs, we performed immunolabeling for PEDF in longitudinal sections of paraffin-embedded whole eyes from mice with 4 weeks of unilateral, microbead-induced ocular hypertension. Qualitatively, elevated IOP increased the overall intensity of PEDF immunlabeling, as compared to saline injection (Figure 3B). Robust immunolabeling of PEDF was noted in vasculature in the OPL (arrows), RGCs in the GCL (filled arrowheads) and Müller cell endfeet in the NFL (open arrowheads), as determined by anatomical location and cytoarchitecture (Figure 3B). Quantification of layer-specific intensities revealed that the intensity of PEDF immunolabeling increased 80% - 3.8-fold across all layers of retina from microbead-injected eyes, as compared to saline-injected eyes (p<0.05 for both; Figure 3C). The greatest increase in labeling was noted in the GCL/NFL (Figure 3C).

### Elevated pressure alters PEDF production by RGCs and Müller cells *in vitro*

**Figure 4:**
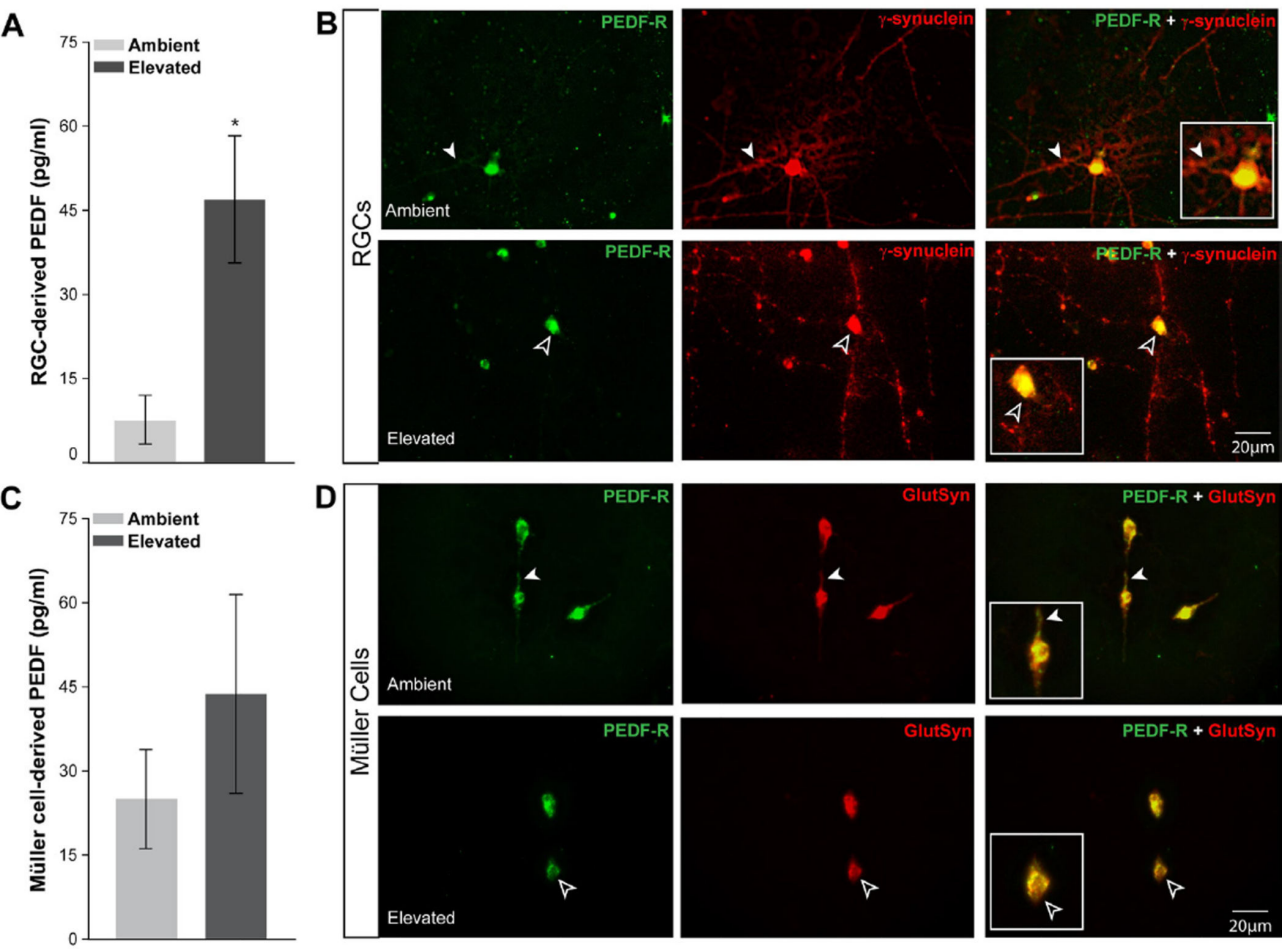
Elevated pressure alters PEDF production and localization in RGCs and Müller cells *in vitro*. **A**. Graphical representation of PEDF concentration (pg/ml; y-axis) in media from primary cultures of purified RGCs exposed to ambient or elevated pressure for 48 hours, as measured by multiplex ELISA. Asterisk denotes p<0.05. **B**. Fluorescent micrographs of PEDF (green) and γ-synuclein (red) immunolabeling in purified, primary cultures of RGCs exposed to either ambient (top panels) or elevated (bottom panels) pressure for 48 hours reveals a reduction in localization of PEDF to neurites following exposure to elevated pressure (black arrowheads) versus ambient pressure (white arrowheads). **C**. Graphical representation of PEDF concentration (pg/ml; y-axis) in media from primary cultures of purified Müller cells exposed to ambient or elevated pressure for 48 hours, as measured by multiplex ELISA. **D**. Fluorescent micrographs of PEDF (green) and glutamine synthetase (red) immunolabeling in purified, primary cultures of Müller cells exposed to either ambient (top panels) or elevated (bottom panels) pressure for 48 hours reveals retraction in cellular processes that is associated with a reduction in PEDF staining following exposure to elevated pressure (black arrowheads), as compared to ambient pressure (white arrowheads).

To validate pressure-induced increases in PEDF by RGCs and Müller cells specifically, we examined PEDF release and localization in cultures of purified RGCs or Müller cells, following 48 hours of ambient or elevated pressure. Since RGCs undergo apoptosis when exposed to elevated pressure, the concentration of PEDF in the culture media was normalized to the density of RGCs [20]. Quantification of PEDF secretion revealed that RGCs release PEDF at ambient pressure *in vitro*, and that exposure to elevated pressure significantly increases PEDF secretion, by 5.4-fold (p<0.05; Figure 4A). Fluorescent immunolabeling indicated that localization of PEDF in RGCs was concentrated in γ-Synuclein+ RGC somas at both ambient and elevated pressure, as evidenced by the yellow appearance of co-labeling (Figure 4B). While PEDF expression was observed in RGC neurites at ambient pressure (white arrowheads, Figure 4B), elevated pressure induces a marked absence of PEDF labeling in RGC neurites as well as a qualitative increase in the intensity of PEDF labeling in RGC somas (Figure 4B).

Müller cells produce PEDF at ambient pressure *in vitro*, and exposure to elevated pressure trends towards increased secretion of PEDF (Figure 4C). However, this difference was not statistically significant (p=0.07; Figure 4C). In a pattern consistent with RGCs, localization of PEDF in Müller cells was concentrated in glutamine synthetase+ Müller cell bodies at both ambient and elevated pressure, as evidenced by the yellow appearance of co-labeling (Figure 4D). While PEDF expression was observed in Müller cell processes at ambient pressure (white arrowheads, Figure 4D), elevated pressure induced both a retraction of Müller cell processes and a subsequent restriction of PEDF labeling to the soma (Figure 4D).

### PEDF-R, like PEDF, is associated with RGCs and Müller cells in healthy retina

**Figure 5:**
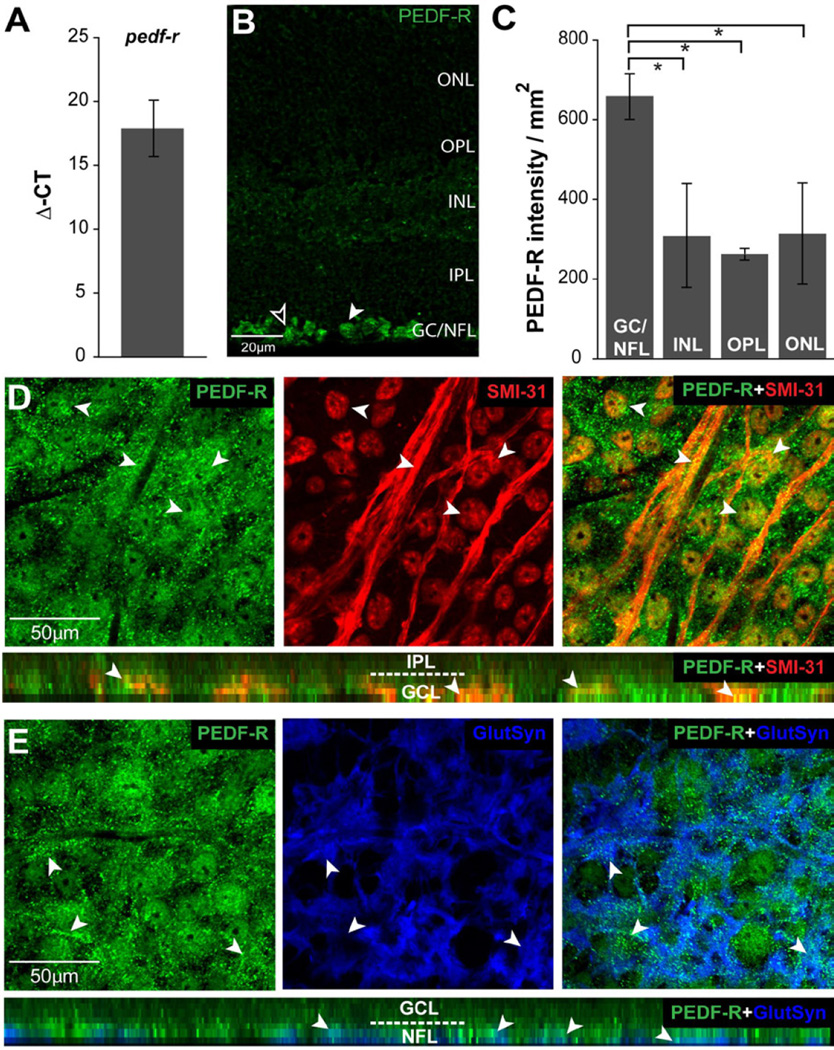
PEDF-R is constitutively expressed by RGCs and Müller cells in healthy retina. **A**. Graphical representation of pedf-r total mRNA levels in naïve C57 retina as measured by qRT-PCR. Y-axis represents the average delta of threshold cycle (Δ-CT) values for pedf-r normalized to the control gene gapdh. **B**. Representative micrograph of longitudinal sections of retina from naïve mice immunolabeled with antibody against PEDF-R. Morphology consistent with localization to RGCs (white arrowheads) and Müller cells (black arrowheads). **C**. Retinal layer-specific quantification of PEDF-R labeling, expressed in intensity (arbitrary units) per area (mm2). All asterisks denote p<0.05. **D**. Representative confocal micrographs with orthogonal view (bottom panel) of wholemount retina from naïve mice co-immunolabeled with antibodies against PEDF-R (green) and the RGC marker SMI-31 (red). PEDF-R localizes to SMI-31+RGC soma and axons, as indicated by the yellow appearance of punctate immunolabeling (arrowheads). **E**. Representative confocal micrographs with orthogonal view (bottom panel) of wholemount retina from naïve mice co-immunolabeled with antibodies against PEDF-R (green) and the Müller cell marker glutamine synthetase (GluSyn; blue). PEDF-R co-localizes with Müller cell endfeet in the NFL, as indicated by the white appearance of immunolabeling (arrowheads). ONL: Outer Nuclear Layer; OPL: Outer Plexiform Layer; INL: Inner Nuclear Layer; IPL: Inner Plexiform Layer; GCL: Ganglion Cell Layer; NFL: Nerve Fiber Layer.

To determine how elevated pressure may alter retinal responses to PEDF, we examined expression of its high-affinity receptor PEDF-R, which exclusively binds PEDF [5]. We first examined expression and localization of PEDF-R in retina from naïve mice. In accordance with previous literature [15,24,25], we detected constitutive expression of the *pedf-r* gene in murine retina (Figure 5A). PEDF-R immunolabeling in paraffin sections revealed that PEDF-R protein localized to all nuclear layers of the retina, as well as the OPL and NFL. However, the intensity of immunolabeling appeared most robust in the GCL and NFL (Figure 5B). Based on cellular morphology and retinal architecture, PEDF-R immunolabeling appeared to be associated with rounded RGC somas in the GCL (white arrowhead) and triangular Müller cell end-feet in the NFL (black arrowhead; Figure 5B), in a pattern resembling PEDF (Figure 5B compared to 1B). Layer-specific quantification of labeling intensity confirmed that PEDF-R immunolabeling was approximately 2-fold higher in the GCL/NFL than in INL, OPL or OPL (p<0.05 for all; Figure 5C).

To confirm PEDF-R localization to RGCs and Müller cells in the GCL and NFL, we performed co-immunolabeling against PEDF-R and cell type-specific markers for RGCs (SMI-31) and Müller cells (glutamine synthetase) in whole-mount naïve retina. PEDF-R immunolabeling was localized in the space surrounding perinuclear labeling of SMI-31 in circular patterns consistent with RGC soma (arrowheads; Figure 5D). Co-localization between PEDF-R and SMI- 31 immunolabeling was also apparent in RGC axons, as indicated by yellow punctae (arrowheads; Figure 5D). Orthogonal sections through our confocal z-stacks confirmed both PEDF-R localization to the GCL and association between PEDF-R and SMI-31 immunolabeling in RGCs (arrowheads; bottom panel; Figure 5D). Similarly, PEDF-R immunolabeling localized in and around glutamine synthetase+ Müller cell end-feet in the NFL, as evidenced by the white appearance of punctate co-labeling (arrowheads, Figure 5E). Orthogonal sections through our confocal z-stacks confirmed PEDF-R localization to the NFL and the GCL as well as co-localization of PEDF-R punctate labeling with glutamine synthetase+ Müller cell end-feet (arrowheads, bottom panel; Figure 5E).

### Elevated IOP increases PEDF-R expression in murine retina

**Figure 6:**
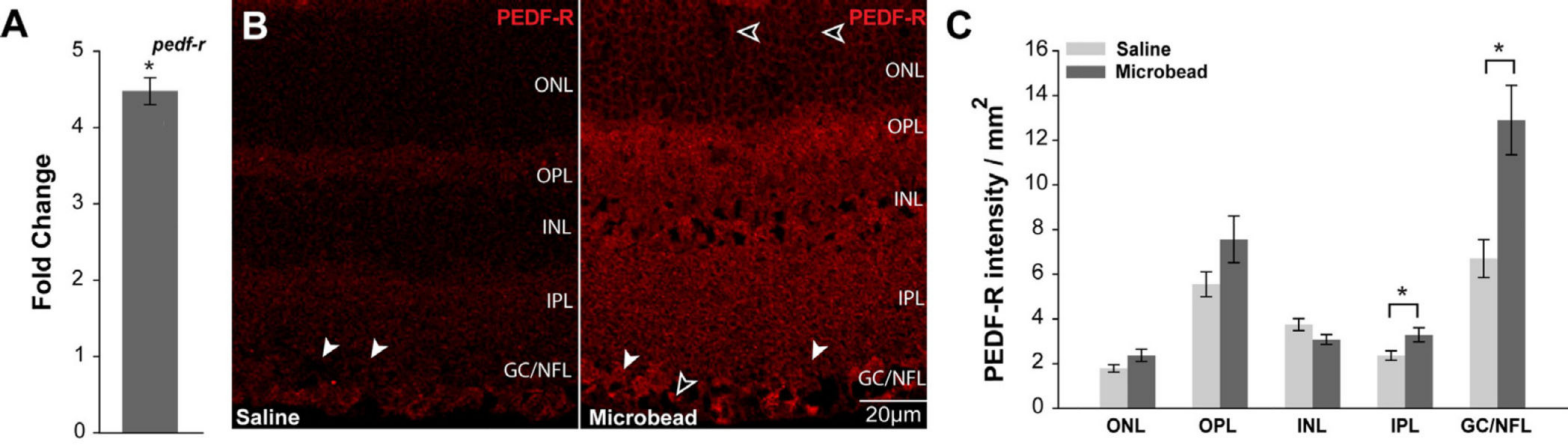
Elevated IOP increases PEDF-R expression in murine retina. **A**. Graphical representation of *pedf-r* total mRNA in retina from saline- and microbead-injected mice, as measured by qPCR. y-axis represents fold change in Δ-CT values for *pedf-r* (normalized to the control gene gapdh) for microbead-injected versus saline-injected. Asterisk denotes p<0.05. **B**. Representative micrographs of immunolabeling for PEDF-R in longitudinal sections of retina from saline- (right panel) and microbead-injected (left panel) mice demonstrate increased intensity of labeling, particularly in the IPL, GCL and NFL, as compared to saline-injected eyes. The pattern of PEDF-R immunolabeling is consistent with RGC soma in the GCL (white arrowheads) and Müller cell processes and endfeet in the ONL and NFL (black arrowheads), respectively. **C**. Retinal layer-specific quantification of PEDF-R labeling, expressed in intensity (arbitrary units) per area (mm^2^). All asterisks denote p<0.05. ONL: Outer Nuclear Layer; OPL: Outer Plexiform Layer; INL: Inner Nuclear Layer; IPL: Inner Plexiform Layer; GCL: Ganglion Cell Layer; NFL: Nerve Fiber Layer

We then sought to determine how elevated IOP alters PEDF-R expression. In mice with 4 weeks of unilateral, microbead-induced glaucoma, we examined retinal expression and localization of PEDF-R. In retina from microbead-injected eyes, *pedf-r* mRNA expression increased by more than four-fold (p<0.05), as compared to retina from normotensive eyes (Figure 6A). There was no significant difference in *pedf-r* expression between naïve and saline-injected conditions (p>0.05).

To determine whether IOP-induced elevations in pedf-r expression are accompanied by alterations in protein localization, we performed immunolabeling for PEDF-R in retinal sections from mice with unilateral, microbead-induced ocular hypertension. The pattern of PEDF-R labeling was similar in retina from naïve and saline-injected eyes, with PEDF-R labeling noted in RGC soma (white arrowheads) and Müller cell endfeet (black arrowheads) in the GCL and NFL, respectively (Figure 6B). However, despite similar levels of pedf-r gene expression and pattern of PEDF-R protein localization, the relative distribution of PEDF-R protein expression between retinal layers differed between retina from naïve mice and retina from saline-injected mice. In particular, saline-injected eyes exhibited less PEDF-R immunolabeling in the INL and IPL than that observed in retina from naïve mice (Figure 6B compared to 5B). In retina from microbead-injected eyes, PEDF-R labeling shared a similar pattern of expression as that observed in retina from naïve eyes, but with greater signal intensity, particularly in the IPL, GCL and NFL (Figure 6B). Additional labeling consistent with Müller cell processes was also apparent in the OPL of retina from microbead-injected eyes (black arrowheads; Figure 6B). To measure changes in observed PEDF expression, we quantified the intensity of immunolabeling per unit area (arbitrary units/mm^2^). In the IPL, PEDF-R expression increased by 32% in retina from microbead-injected eyes, as compared to retina from saline-injected eyes (p<0.05; Figure 6C). As with PEDF expression, PEDF-R expression increased the most in the GCL/NFL of retina from microbead-injected eyes, as compared to saline-injected eyes (2-fold; p<0.05; Figure 6C).

### Elevated pressure alters PEDF-R expression by RGCs and Müller cells *in vitro*

**Figure 7:**
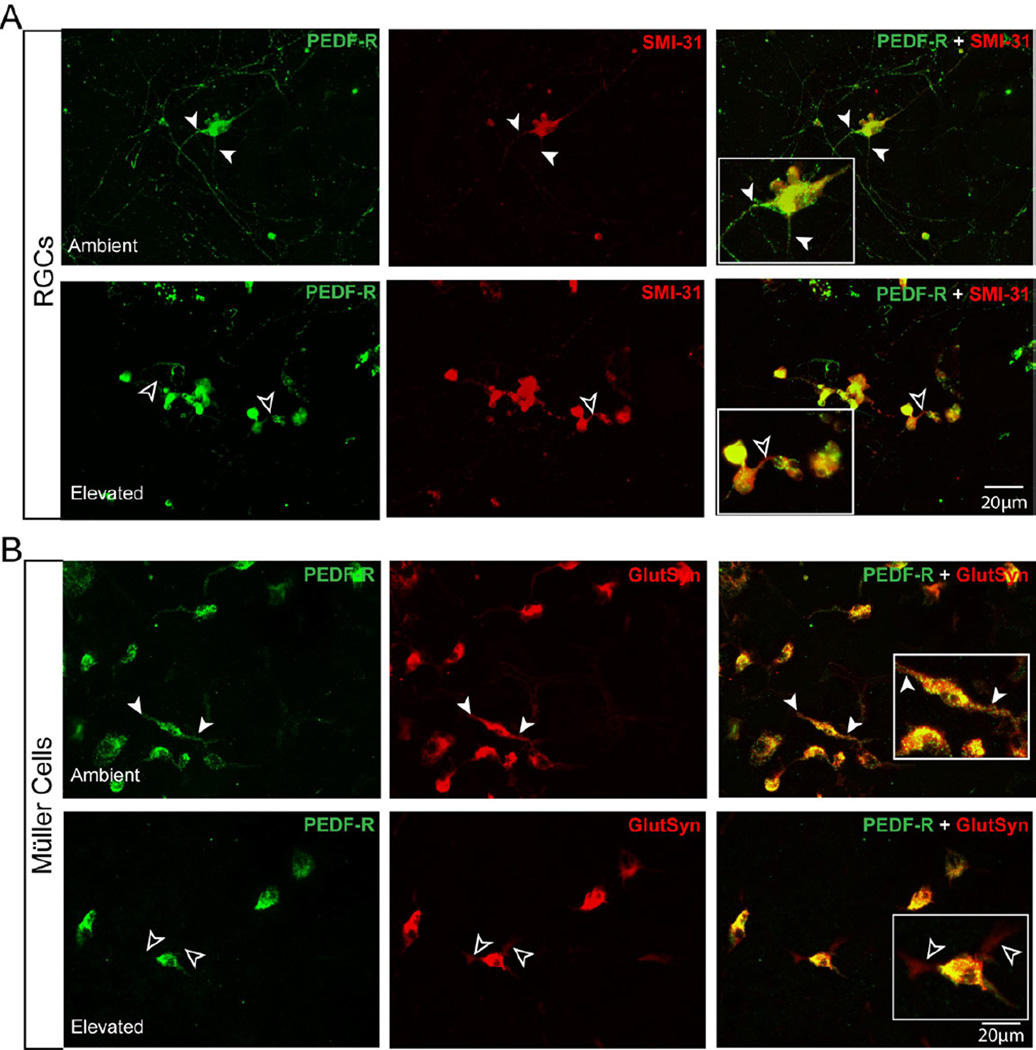
Elevated pressure alters PEDF-R localization in RGCs and Müller cells *in vitro.* **A**. Fluorescent micrographs of PEDF-R (green) and SMI-31 (red) immunolabeling in purified, primary cultures of RGCs exposed to either ambient (top panels) or elevated (bottom panels) pressure for 48 hours reveals a reduction in localization of PEDF-R to neurites (black arrowheads) following exposure to elevated pressure, as compared to ambient pressure (white arrowheads). **B**. Fluorescent micrographs of PEDF-R (green) and glutamine synthetase (red) immunolabeling in purified, primary cultures of Müller cells exposed to either ambient (top panels) or elevated (bottom panels) pressure for 48 hours reveals a reduction in localization of PEDF-R to processes that is associated with process retraction (black arrowheads) following exposure to elevated pressure (black arrowheads) versus ambient pressure (white arrowheads).

To validate pressure-induced changes in PEDF-R expression by RGCs and Müller cells specifically, we examined PEDF-R localization in primary cultures of purified RGCs and Müller cells, following 48 hours of ambient or elevated pressure. Fluorescent immunolabeling revealed that localization of PEDF-R in RGCs was concentrated in SMI-31+RGC somas at both ambient (Figure 7A) and elevated pressure (Figure 7A), as evidenced by the yellow appearance of co-labeling. While PEDF-R expression was observed in RGC neurites at ambient pressure (white arrowheads, Figure 7A), elevated pressure induced a marked absence of PEDF-R labeling in RGC neurites (Figure 7A. Moreover, elevated pressure results in more pronounced PEDF-R expression on the border of RGC somas (white arrowheads, Figure 7A).

Localization of PEDF-R was observed on the soma of glutamine synthetase+ Müller cells at both ambient and elevated pressure, as evidenced by the yellow appearance of punctate co-labeling (Figure 7B). However, like PEDF-R localization in RGCs, the robust localization of PEDF-R to Müller cell processes noted at ambient pressure (white arrowheads, Figure 7B) was not evident at elevated pressure (Figure 7B). This reduction of PEDF-R immunolabeling was accompanied by a retraction of Müller cells processes. However, some glutamine synthetase+ processes remained visible and lacked PEDF-R labeling (white arrows; Figure 7B).

### PEDF signaling inhibits pressure-induced apoptosis of RGCs

**Figure 8:**
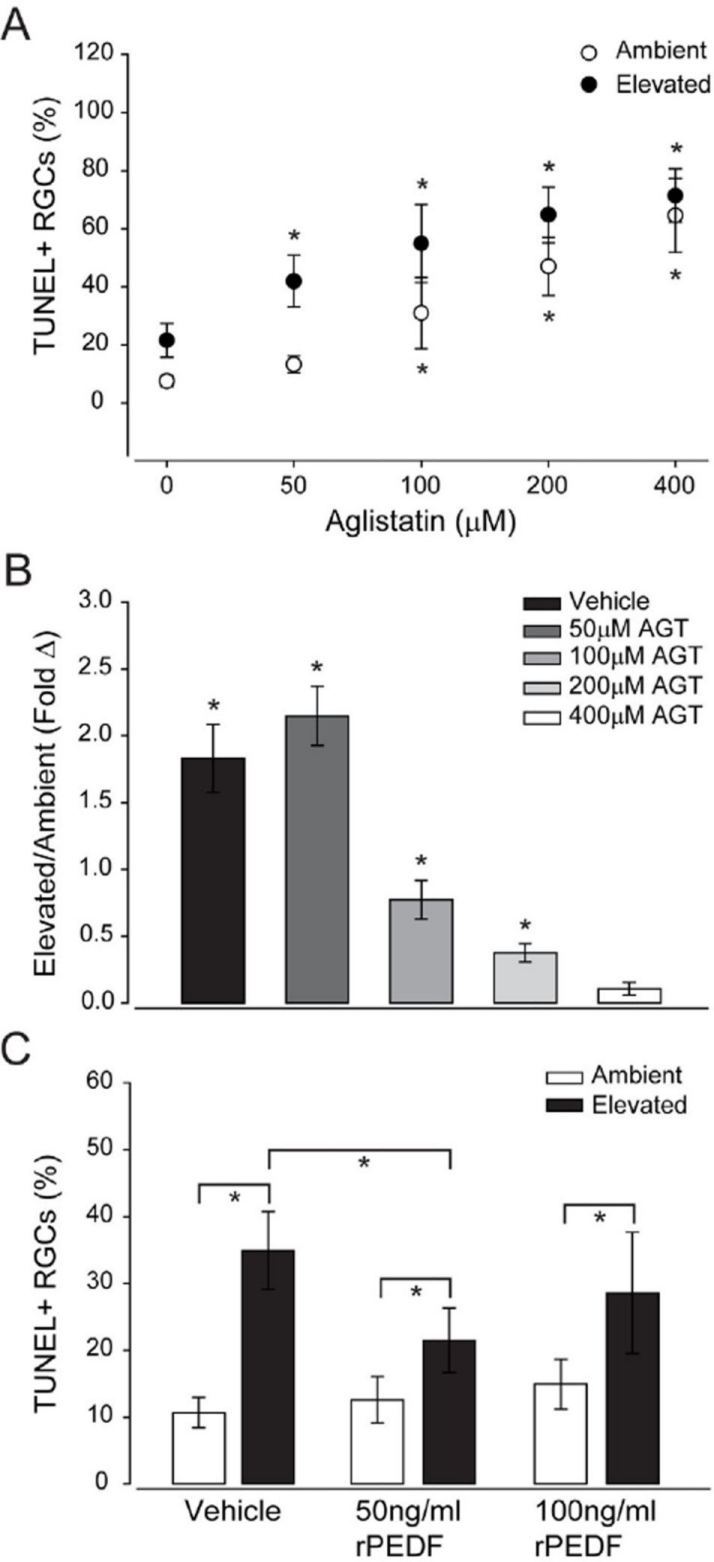
PEDF signaling protects RGCs from pressure-induced apoptosis. **A**. Graphical representation of the percentage TUNEL+RGCs treated with vehicle or the PEDF-R specific inhibitor aglistatin (50 µM, 100 µM, 200 µM, 400 µM) and exposed to ambient or elevated pressure for 48 hours. Asterisks denote statistical significance (p<0.05) between vehicle and drug treatments within each pressure condition. **B**. Graphical representation of the same data in (A) depicted as the percentage change in TUNEL+RGCs between ambient and elevated pressure within each drug treatment. Asterisks denote statistical significance between ambient and elevated pressure within each drug treatment (p<0.05). **C**. Graphical representation of the percentage of TUNEL+RGCs treated with vehicle, 50ng/ml recombinant PEDF (rPEDF) or 100 ng/ml rPEDF and exposed to ambient or elevated pressure for 48 hours. Asterisks denote statistical significance (p<0.05).

To assess the relevance of PEDF signaling to RGC viability, we exposed primary cultures of purified RGCs to ambient or elevated pressure for 48 hours in the presence or absence of the selective inhibitor of PEDF-R atglistatin or recombinant PEDF [22]. TUNEL labeling revealed that PEDF-R inhibition with atglistatin induced RGC apoptosis in a dose-dependent manner at both ambient and elevated pressure (Figure 8A). At ambient pressure, application of 50 µM – 400 µM atglistatin increased the percentage of TUNEL+ RGCs by 2.5–5.5- fold, as compared to vehicle treatment (p<0.05 for all; Figure 8A). At elevated pressure, treatment with 50 µM – 400 µM atglistatin increased the percentage of TUNEL+ RGCs by 4–6-fold, as compared to vehicle treatment (p<0.05 for all; Figure 8A). Consistent with previous findings [3,21], exposure to 48 hours of elevated pressure increased apoptosis of RGCs by 2-fold (Figures 8B,C). At elevated pressure, 50 µM, 100 µM and 200 µM doses of atglistatin increased apoptosis of RGCs by 2.5-fold, 77% and 37%, respectively, as compared to the same doses at ambient pressure (p<0.05 for both; Figure 8B). At the 400 µM dose, apoptosis of RGCs was similar for both ambient and elevated pressure conditions (p>0.05; Figure 8B), suggesting toxicity of PEDF-R inhibition at our highest dose. When treated with 50 ng/ml or 100 ng/ml recombinant PEDF (rPEDF), basal levels of RGC apoptosis at ambient pressure were unchanged (Figure 8C). In contrast, treatment with 50 ng/ml rPEDF reduced pressure-induced apoptosis of RGCs by 38%, as compared to vehicle treatment (Figure 8C). Treatment with 100 ng/ml rPEDF did not alter the percentage of TUNEL+RGCs, as compared to vehicle treatment (Figure 8C).

## Discussion

Here we describe constitutive and pressure-related in the expression and localization of PEDF and its high-affinity receptor PEDF-R in retina and examined the relevance of PEDF signaling to pressure-induced apoptosis of RGCs. Both PEDF and its receptor are constitutively expressed in healthy retina, preferentially localizing to the ganglion cell and nerve fiber layers. The expression of PEDF and PEDF-R in the inner retina suggests that PEDF signaling is highly localized and site-specific. Moreover, PEDF and PEDF-R were upregulated in retina with microbead-induced ocular hypertension, suggesting that PEDF signaling may be particularly relevant for glaucoma pathophysiology. Elevated IOP, the primary modifiable glaucomatous risk factor, increased expression of both PEDF and PEDF-R without altering the pattern of expression in the inner retina. However, it is interesting to note that elevated IOP induced expression of PEDF in the outer plexiform layer (OPL), suggesting that PEDF signaling could be relevant for outer retinal neurons and/or retinal vasculature of the OPL in glaucoma. The latter would not be surprising, given the anti-angiogenic properties of PEDF in the retina and elsewhere [7,26]. Although we are the first to describe increased expression of PEDF and PEDF-R in an induced model of ocular hypertension, our findings are consistent with previous studies that have established a role for PEDF signaling in RGC degeneration induced by ischemia [13], trophic factor withdrawal [5], and inherited ocular hypertension [11].

A key objective underlying this study was to identify the specific retinal cell types that express PEDF and PEDF-R. Our *in vivo* data suggest that PEDF and PEDF-R expression is associated with RGCs and Müller cells in the GCL and NFL. Our *in vitro* data in purified primary cultures of RGCs and Müller cells confirmed expression of PEDF and PEDF-R by both of these cell types. These results corroborate earlier findings that describe a constitutive signaling pathway where Müller cell end-feet secrete PEDF that bind to receptors on nearby RGC cell somas [13]. This paracrine mechanism is proposed to exert neuroprotective effects, attenuating glaucomatous pathology in diseased retinas. However, our data suggest that PEDF could function as an autocrine or paracrine signal in the inner retina, in which RGC-or Müller cell-derived PEDF could bind PEDF-R on the same cell or on neighboring cells.

We further confirmed pressure-induced elevation in PEDF expression noted *in vivo* with our *in vitro* studies and determined that PEDF secretion by RGCs increases in response to elevated hydrostatic pressure. In contrast, the apparent trend towards increased PEDF secretion by Müller cells exposed to elevated pressure failed to reach statistical significance, suggesting that PEDF responses may be more robust in RGCs than in Müller cells. Given the potential for paracrine PEDF signaling between RGCs and Müller cells, there is a possibility that upregulation of PEDF by Müller cells is secondary to upregulation by RGCs *in vivo*.

Although elevated IOP increases PEDF and PEDF-R expression *in vivo*, it does not appear to alter the global localization pattern of PEDF or PEDF-R. However, our *in vitro* data suggests that elevated pressure may alter the subcellular localization. At ambient pressure both PEDF and PEDF-R localized to the cell bodies and processes of RGCs and Müller cells. However, elevated pressure significantly reduced localization of PEDF and PEDF-R in neurites of RGCs and processes of Müller cells. In both cases, this pressure-induced shift in protein localization accompanied retraction and thinning of processes, but was not attributable to complete loss of these structures. Given the similarities in protein localization between PEDF and PEDF-R, it is likely that the localization pattern observed for PEDF results from labeling of PEDF bound to the receptor.

Finally, we determined the functional relevance of PEDF signaling to RGC survival *in vitro*, using gain and loss of function studies. Inhibition of PEDF signaling with the potent and selective inhibitor of PEDF-R atglistatin [29] resulted in a significantly higher percentage of TUNEL+ RGCs, regardless of pressure condition at all but our lowest dose tested. This suggests that PEDF signaling promotes constitutive survival of RGCs, at least *in vitro*. Despite its constitutive effects, aglistatin, further increased the percentage of TUNEL+ RGCs at elevated pressure for all but the highest dose tested. The converse gain of function studies, in which RGCs were treated with rPEDF, revealed a reduction in pressure-induced apoptosis of RGCs. That this neuroprotective effect was noted only at the lowest dose tested, suggests that the neuroprotective benefits of PEDF could be concentration-dependent. Together, these data suggest that PEDF signaling can directly modulate pressure-induced apoptosis in RGCs, which aligns well with previous studies suggesting a neuroprotective role for PEDF in glaucoma [19,21].

While our experiments imply neuroprotective roles for PEDF signaling, we did not elucidate any downstream cascades that may occur after PEDF binds to PEDF-R. Literature suggests that PEDF acts via the Ras mitogen-activated protein kinase (Ras-MapK) pathway [27,28]. Activated Ras phosphorylates MapK [18], an upstream modulator of various extracellular-signal regulated kinases (ERKs) which ultimately regulate gene expression. However, PEDF may act via other signaling pathways, as PEDF-R reveals a patatin-like phospholipase domain at its C-terminus [25] and possesses phospholipase A2 activity by cleaving fatty acids from phospholipids [15]. These findings suggest that PEDF-R behaves similarly to phospholipase C in the IP3-DAG signaling cascade, in which products of phospholipid cleavage become downstream second messengers. Indeed, the neuroprotective qualities of PEDF may be mediated by pathways akin to canonical IP3-DAG signaling. It is important to remember, though, that PEDF is a promiscuous ligand; for example, secreted PEDF has been shown to bind a variety of scaffolding proteins including hyaluronan [29] and other glycosaminoglycans [30]. Clearly, PEDF acts on a multitude of signaling cascades and exerts pleiotropic effects; it is possible that the neuroprotective and anti-angiogenic effects of PEDF are mediated by separate pathways.

Overall, our findings suggest that changes in constitutive PEDF signaling accompany glaucomatous pathology, particularly in the inner retina. Furthermore, glaucoma-related increases in PEDF signaling may serve an inherent, neuroprotective function for RGCs that is initiated by RGCs themselves.

## Figures and Tables

**Table 1:** Primary antibodies used for immunohistochemistry and immunocytochemistry. Primary antibodies, sources, and concentrations used for detection of PEDF, PEDF-R and labeling of RGCs and Müller cells in murine retina.

Target	Antibody name	Concentration used	Vendor	Catalog #
PEDF	Mouse anti-human PEDF	20 µg/ml	Abcam (Cambridge, MA)	115489
PEDF-R	Goat anti-human PEDF-R/PNPLA2	20 µg/ml	R&D Systems (Minneapolis, MN)	AF5387
	Goat anti-human Adipose Triglyceride Lipase	2.5 µg/ml	Abcam (Cambridge, MA)	85858
Retinal ganglion cell (RGC)	Mouse anti-mammalian SMI-31 (phosphorylated neurofilaments)	1 µg/ml	Covance (Princeton, NJ)	SMI-31R
	Rabbit anti-mammalian Gamma Synuclein	10 µg/ml	Abcam (Cambridge, MA)	55424
Müller Cells	Goat anti-human Glutamine Synthetase	4 µg/ml	Santa Cruz Biotechnologies (Santa Cruz, CA)	Sc-6640
	Rabbit anti-human Glutamine Synthetase	4 µg/ml	Santa Cruz Biotechnologies (Santa Cruz, CA)	Sc-6640-R
